# Acute and Sub-chronic Oral Toxicity Study of Ammonium Persulfate in Spraque-Dawley Rats

**DOI:** 10.5487/TR.2009.25.3.132

**Published:** 2009-09-01

**Authors:** Yong Soon Kim, Min Won Baek, Jae Hyuck Sung, Hyun Youl Ryu, Jin Sik Kim, Hyun Sun Cho, Byung Gil Choi, Min Sub Song, Moon Yong Song, Eun Ju Baik, Young Kuk Choi, Jong Kyu Kim, Il Je Yu, Kyung Seuk Song

**Affiliations:** 14 Department of Toxicopathology, Korea Environment & Merchandise Testing Institute, Songdo-dong 7–44, Yeonsu-gu, Incheon, 406-840 Korea; 24 Korea Occupational Safety and Health Agency, Daejeon, 305-380 Korea; 34 Fusion Technology Research Institute, Hoseo University, Chungnam, 336-795 Korea

**Keywords:** Ammonium persulfate, Acute & sub-chronic toxicological test, SD rats, GHS, NOAEL

## Abstract

The toxicity test of ammonium persulfate was conducted to ensure of its potential toxic effects according to the single-dose acute oral toxicity study (OECD Guideline 423) and 90-day repeated dose sub-chronic oral toxicity study guideline (OECD Guideline 408) for establishing national chemical management system, and matching in the Globally Harmonized Classification System (GHS) category. In acute oral toxicity study, pasty stool, perineal contamination and temporary body weight decrease were observed after dosing 1st and 2nd challenge (300 mg/kg body weight). All test animals were dead within 6 hours after dosing at 3rd challenge (2000 mg/kg body weight). Therefore, the GHS class of test substance is considered class 4. In sub-chronic toxicity study, body weight changes, food consumptions, hematological, biochemical and pathological examination did not show any noticeable and significant differences between the administered (5, 20, 80 mg/kg body weight) and control (vehicle only) group animals. Based on these results, the no observed adverse effect level (NOAEL) is considered above 80 mg/kg body weight.

## INTRODUCTION

Ammonium persulfate is used for hair dyeing dye, bleaching, hair dyeing medicine, bleaching agent for food starch and cosmetics such as skin care medicine (Lewis, 1997; Nikitakis *et al.*, 1990). Food and drug association (FDA) was presented that ammonium persulfate was used in 30 cosmetics product in 1998 (FDA, 1998). And ammonium persulfate is used for a reductant, a restrainer at photo print, industrial starch modifier, preserving foods, copper oxidizing agent, and electronic circuit board such as industrial field (Merget *et al.*, 1999; Pang *et al.*, 2001; Wenninger *et al.*, 2000).

The National Statistical Office of North America, Europe and Japan stated that persulfate materials such as potassium persulfate and sodium persulfate including ammonium persulfate were produced at amount of 65,400 tons per year (RTEC, 1994) and about 10,000 tons in China. And in Korea, the amount is reached 1,155,220 kg per year according to the 15 company data (UNEP, 2005).

However, ammonium persulfate may cause an unexpected health effect such as immediate and prolonged skin reactions including irritant dermatitis, allergic eczematoid dermatitis, local contact rash, systemic rash, rhinitis, asthma and fainting though out the exposure of aerosol or vapor skin contact and inhalation to people dealing with this material in many quarters (Mensing *et al.*, 1998; Merget *et al.*, 1999; Vanjoost *et al.*, 1991) And there was a case report about the actual work environment (Gamboa *et al.*, 1999), but its incidences were not well known and the incidental rate of toxic symptoms induced by ammonium persulfate also yet.

We carried out acute and sub-chronic toxicity test with ammonium persulfate using SD rats through the OECD guidelines to provide exact toxicological information and to sort out its GHS category, hazard prediction and management system proposal for the establishment of materials safety and public health.

## MATERIALS AND METHODS

***Test substance.*** Ammonium persulfate (Purity, 99.4%; CAS No. 7727-54-0; Lot number, 126K0150) was obtained from Sigma-Aldrich (USA), and kept at room temperature. For vehicle, distilled water was used base on the characteristics of test substance solubility analysis.

***Animals.*** Specific pathogen free (SPF) Sprague-Dawley (SD) rats were purchased from ORIENT BIO Inc. (Korea) for acute (n = 9; Only female) and for sub-chronic toxicity test (n = 80; 40 males and 40 females), and acclimated for 7 days before starting the experiments. During the acclimation and experimental periods, the rats were housed in polycarbonate cages (2 rats per cage) in animal room with controlled temperature (22 ± 3°C) and humidity (50 ± 20 %), and a 12-hr light/dark cycle. The rats were fed rodent chow (Harlan Teklad, USA) and filtered tap water *ad libitum*. On the dosing day, the rats were 7 (for acute) or 6 (for sub-chronic) week old ranging 150~180 g for female and 190~220 g for male. Acclimation and all animal experiments were approved by the Institute animal use and care committee of KEMTI. (Approval number: 2008-94-1162).

***Study of acute oral toxicity.*** The study was conducted in accordance with OECD guidelines for the Testing of Chemical No. 423 'Acute Toxic Class method' (as revised in 2001) and the Notification of the Ministry of Labor No. 2008-11 'Examination of toxic risk about industrial chemicals' (as revised in 2008). 3 female rats were assigned randomly and had fasted for approximately 12 h prior to dosing. And the test substance was formulated as a 30 mg/ml suspension in distilled water then administered to each animal at a dose of 300 mg/kg body weight by oral gavage and the same procedure repeated once more for reconfirmation (1st, 2nd step). Since there were no deaths during the two preliminary studies, the oral dose of 2,000 mg/kg (200 mg/ml distilled water suspension) was set for the last challenge using remains of 3 female rats 1 wk after the preliminary (3rd step). Each animal was observed for signs of toxicity and behavioral changes at 1 and 3 hr after test substance administration and once daily thereafter for 14 days. Mortality and morbidity checks were performed daily. Body weights were recorded before administration, at the day 1, 3, 7, and at termination. On day 15, survived rats were euthanatized, necropsies were performed, and any gross observations were recorded.

***Study of sub-chronic toxicity.*** A repeated-dose 90-day oral toxicity study was conducted in accordance with OECD guidelines for the Testing of Chemical No. 408 'Repeated Dose 90-day Oral toxicity Study in Rodents' (as revised in 1998) and the notification of the Ministry of Labor No. 2008-11 'Examination of toxic risk about industrial chemicals' (as revised in 2008). Briefly, all animals had fasted for approximately 5 h prior to dosing. Groups of 10 randomly assigned rats of each sex were received distilled water suspended 5, 20 and 80 mg test substance/kg/body weight/day per oral daily for 90-day. The doses were selected based on the results of the acute toxicity study. The control groups received vehicle (filtered tap water) only. After dosing, food and water were provided *ad libitum*. All animals were subjected to ophthalmologic examination prior to administration and at study termination and examined daily for any visible signs of effects, mortality and morbidity of administration. All were weighed once during acclimatization, one day prior to dosing, and weekly for experimental periods. Food consumed by animals was recorded over 5 day in each week.

***Clinical examination:*** Overnight urine samples were collected from all animals under food and water deprivation once the day before necropsy for clinical pathology, rats were placed in metabolism cages and urine collected for subsequent analysis. Urinalysis included quality, color, clarity, volume, pH, glucose, specific gravity, protein, ketone, bilirubin, blood, urobilinogen, and microscopic urine sediment examination. At the termination of the study, animals were anesthetized with $CO_2$, gas inhalation and blood samples were collected from the descending aorta, collected in heparinized vacutainers, and analyzed for ALB (albumin), ALP (alkaline phosphatase), Ca (calcium), CHO (cholesterol), CRE (creatinine), gamma-GT (gamma-glutamyl transpeptidase), GLU (glucose), AST (aspartate aminotransferase), ALT (alanine aminotransferase), LDH (lactate dehydrogenase), MG (magnesium), TP (total protein), UA (uric acid), BUN (blood urea nitrogen), T-BIL (total bilirubin), IP (inorganic phosphorus), TG (triglyceride), CPK (creatine phosphokinase), Na (sodium), K (potassium) and Cl (chloride) using a biochemical blood analyzer (Hitachi 1780, Hitachi, Japan). The blood was also analyzed for the WBC (white blood cell count), RBC (red blood cell count), Hb (hemoglobin concentration), HTC (hematocrit), MCV (mean corpuscular volume), MCH (mean corpuscular hemoglobin), MCHC (mean corpuscular hemoglobin concentration), RDW (red cell distribution width), PLT (platelet counts), MPV (mean platelet volume), NE (neutrophils), LY (lymphocytes), MO (monocytes), EO (eosinophils) and BASO (basophils) using a blood cell counter (Hemavet 0950, CDC Tech., USA).

***Organ weights and histopathology:*** After collecting the urine and blood, all animals were conducted to euthanasia by $CO_2$ gas and necropsies were performed. The gross observations and organ weights of lungs, trachea, brain, spinal cord, salivary glands, thymus, heart, sternum with bone marrow, adrenals, liver, spleen, kidneys, thyroid/parathyroid, urinary bladder, ovaries and fallopian tubes, uterus, vagina, esophagus, prostate and seminal vesicles, peripheral nerve (sciatic), stomach, small & large intestine, mesenteric and mandibular lymph nodes, pancreas, pituitary, aorta, mammary gland, harderian gland, skin, nasal turbinates, skeletal muscle, epididyma, testes, and eyes were recorded and removed carefully for fixation. Tissues were fixed in 10% neutral buffered formalin, embedded in paraffin, sectioned, stained with hematoxylin & eosin, and examined microscopically.

***Statistical analysis.*** GHS class was calculated through the OECD guideline 423 (OECD, 2001) in the acute toxicity test. The results are presented as the mean ± standard deviation (S.D.). The differences parameters (body weights, organ weights, and the results of the urine & blood biochemistry and hematology) studied between the groups in the acute and sub-chronic toxicity test were assessed by the standard two-way analysis of variance (ANOVA). If these showed statistical significance, Duncan's or Dunnett's multiple range test were used to compare (SPSS for Windows 12.0 K). *P*-values < 0.05 were considered as statistically significance.

## RESULTS

***Study of acute toxicity.*** There were no deaths of rats administered 300 mg/kg of ammonium persulfate (1st, 2nd Step), the clinical signs include pasty stool, perineal contamination and body weight decreasing were observed. However, these were temporal and recovered in the observation periods. Additionally, no gross lesions were found in any of the organs at necropsy (data not shown).

Because of no deaths founded during the two steps, the oral dose of 2,000 mg/kg was set for the last step. All administered animals showed hunched back position and died within 6 hr after dosing. Symptoms of diarrhea, including discoloration to dark-red in forestomach & cecum and intestinal fluids filling, discoloration in the lung and marginal legion of liver were also founded at necropsy.

Based on these results of acute oral toxicity tests, ammonium persulfate was calculated as GHS 4 ($LD_{50}$ cut-off: 500 mg/kg body weight) class substance. And the doses for sub-chronic toxicity test were determined.

***Study of sub-chronic toxicity.*** Sub-chronic oral administration of ammonium persulfate at dose of 5, 20 and 80 mg/kg for 90 days produced no signs of toxicity or gross behavioral changes. All rats survived to the end of the 90-day period. No abnormal clinical observations were observed.

Changes in body weight of rats administered with ammonium persulfate, for 90 days, compared to the control group, are illustrated in Fig. 1. Mean body weights of

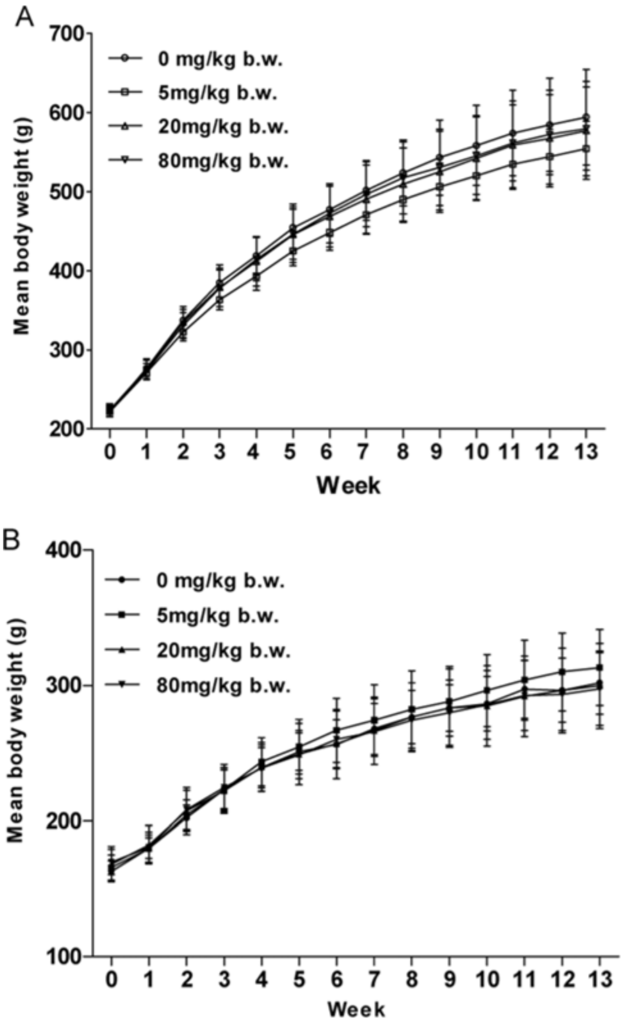

**Fig. 1.** Mean (± S.D.) body weight of males (A) and female (B) rats treated for 90 days with water or suspensions of ammonium persulfate in distilled water at dose levels of 0, 5, 20 and 80 mg/kg day, 7 days/week for 90 days. The rats exposed to the showed no significant differences as compared to the control.

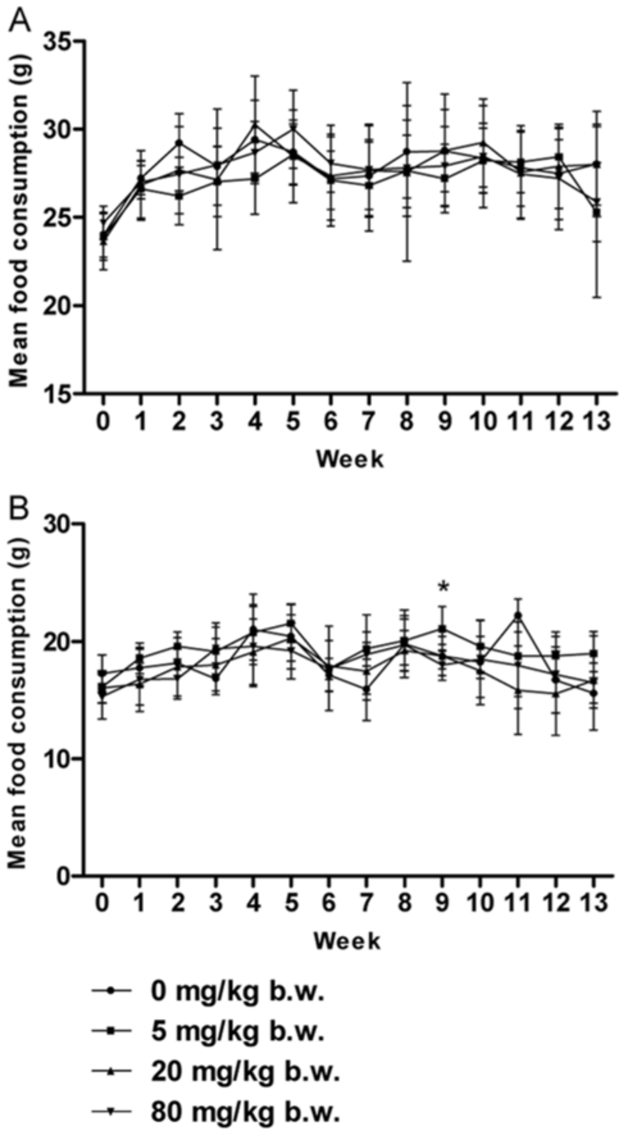

**Fig. 2.** Daily mean (± S.D.) food consumption of male (A) and female (B) rats treated ammonium persulfate for 90 days. *: Significant difference from the control group of female rats ($P < 0.05$). Results are given as mean ± SD, n = 5.

both sex animals in the four groups measured at weekly intervals were not significantly different. However, there was a significant difference about daily mean food consumption (21.08 ± 1.90 *vs* 18.75 ± 1.04 g) between the 5mg/kg-administered and control female rats at 9-week, but these were transitional phenomena (Fig. 2).

***Clinical examination:*** Haematological findings (Table 1) did not show any administration-related effects. No significant differences were seen between the administered and control animals in WBC, RBC, hemoglobin, hematocrits, mean corpuscular volume, mean corpuscular hemoglobin, mean corpuscular hemoglobin concentration, and red cell distribution width. Similarly, platelet, neutrophil, eosinophil, basophil and leukocyte counts in the administered groups were not significantly different from those in the control group. Monocyte count and mean platelet volume were significantly lower ($P < 0.05$) than in controls compared to the female 20 and 80 mg/kg administered group. However, these changes were considered normal physiologic range of SD rats (Matsuzawa *et al*., 2000).

The effects of sub-chronic ammonium persulfate administration on blood chemistry are summarized in Table 2. Administration with ammonium persulfate for 90 days had no significant effect on most parameters of blood chemistry including carbohydrate metabolism, liver and kidney function (Table 2). Increased total protein and decreased blood urea nitrogen, chloride and alkaline phosphatase in male ammonium persulfate - administered groups had a significance ($P < 0.05$). In female, 5 mg/kg administered animals showed increasing lactate dehydrogenase and also considered normal (Matsuzawa *et al*., 2000).

***Organ weights and histopathology:*** No significant differences are shown in the mean weights of brain, liver, kidneys, and *etc*. in the administered and control animals (Table 3). However, there was a significant difference in the 5 mg/kg administered group mean absolute right adrenal gland (0.041 ± 0.005 *vs* 0.031 ± 0.006 g) and in the 20 mg/kg administered group left ovary weights (0.07 ± 0.01 *vs* 0.06 ± 0.001 g) between administered and control female animals.

Microscopic examination did not reveal any administration or dose-related changes. Non-specific histopathological changes of a slight to mild grade inflammation in liver, kidneys and lung, mild vaoculation of liver were found in some animals (of all groups). All changes observed were about equally distributed between the controls and the groups given the test substance (Fig. 3).

## DISCUSSION

The present study has demonstrated that, acute and sub-chronic 90 days oral toxicity of ammonium persulfate according to the OECD guidelines. No major toxicology-related abnormal changes except alimentary tract and death were shown in dose of 300 mg/kg administered rats. But, In 2,000 mg/kg, all animals died after within 6 hr. these results for the test substance as GHS 4 class has been reckoned, and non-toxic amount of 80 mg/kg was set for later test. In sub-chronic toxicity, Ammonium persulfate administration to rats at doses between 0 to 80 mg/kg caused no animal death and prominent signs of toxicity based on clinical examination, urinalysis,

**Table 1.** Mean haematological values in rats given ammonium persulfate orally for 90 days

| Haematologic parameters | Ammonium persulfate (mg/kg) Female (n = 10) | | | | Ammonium persulfate (mg/kg) Male (n = 10) | | | |
|---|---|---|---|---|---|---|---|---|
| | 0 | 5 | 20 | 80 | 0 | 5 | 20 | 80 |
| WBC (K/μl) | 6.93 ± 2.40 | 6.66 ± 2.23 | 6.39 ± 2.35 | 6.43 ± 1.60 | 11.96 ± 2.57 | 12.15 ± 3.05 | 11.11 ± 3.23 | 11.35 ± 2.79 |
| RBC (M/μl) | 8.00 ± 0.46 | 8.22 ± 0.49 | 5.14 ± 0.86 | 7.98 ± 0.48 | 8.30 ± 0.45 | 8.50 ± 0.53 | 8.64 ± 0.55 | 8.46 ± 0.51 |
| Hb (g/dl) | 16.68 ± 0.67 | 17.08 ± 0.93 | 16.72 ± 0.55 | 16.95 ± 0.55 | 16.86 ± 0.80 | 16.98 ± 1.10 | 17.32 ± 0.85 | 16.86 ± 0.99 |
| HCT (%) | 42.31 ± 1.63 | 42.75 ± 1.97 | 42.45 ± 1.89 | 43.14 ± 1.55 | 40.78 ± 2.66 | 41.39 ± 2.74 | 41.64 ± 1.93 | 40.61 ± 2.11 |
| MCV (fl) | 52.98 ± 2.15 | 52.08 ± 1.59 | 52.29 ± 2.87 | 52.97 ± 1.91 | 49.13 ± 1.87 | 48.71 ± 2.34 | 48.29 ± 1.89 | 48.05 ± 1.71 |
| MCH (pg) | 20.87 ± 0.61 | 20.79 ± 0.67 | 20.60 ± 1.11 | 20.81 ± 0.74 | 20.34 ± 0.90 | 19.99 ± 0.90 | 20.07 ± 0.90 | 19.95 ± 0.82 |
| MCHC (g/dl) | 39.43 ± 0.74 | 39.96 ± 0.94 | 39.42 ± 0.81 | 39.30 ± 0.73 | 41.39 ± 1.13 | 41.03 ± 0.59 | 41.60 ± 0.74 | 41.51 ± 1.10 |
| RDW (%) | 20.58 ± 1.09 | 20.73 ± 1.00 | 20.90 ± 1.11 | 20.65 ± 1.40 | 19.14 ± 1.53 | 19.99 ± 2.54 | 20.10 ± 1.89 | 19.80 ± 1.86 |
| PLT (K/μl) | 813.80 ± 97.94 | 747.60 ± 37.72 | 772.90 ± 43.56 | 784.10 ± 69.85 | 741.90 ± 61.75 | 754.50 ± 85.99 | 769.30 ± 71.90 | 787.00 ± 65.71 |
| MPV (fl) | 6.20 ± 0.37 | 6.21 ± 0.44 | 5.83* ± 0.43 | 5.69* ± 0.27 | 5.86 ± 0.31 | 5.98 ± 0.24 | 8.92 ± 0.23 | 5.86 ± 0.26 |
| NEU (K/μl) | 1.77 ± 0.79 | 1.67 ± 0.55 | 1.47 ± 0.52 | 1.43 ± 0.46 | 3.85 ± 0.85 | 3.71 ± 1.00 | 3.61 ± 1.26 | 3.71 ± 1.84 |
| LYO (K/μl) | 4.69 ± 1.85 | 4.56 ± 1.67 | 4.62 ± 1.89 | 4.69 ± 1.47 | 7.28 ± 2.30 | 7.68 ± 2.24 | 6.73 ± 1.78 | 6.97 ± 2.10 |
| MONO (K/μl) | 0.42 ± 0.15 | 0.38 ± 0.18 | 0.28** ± 0.11 | 0.26** ± 0.12 | 0.80 ± 0.22 | 0.73 ± 0.16 | 0.73 ± 0.32 | 0.62 ± 0.23 |
| EOS (K/μl) | 0.03 ± 0.02 | 0.02 ± 0.02 | 0.01 ± 0.01 | 0.10 ± 0.01 | 0.04 ± 0.03 | 0.03 ± 0.04 | 0.04 ± 0.04 | 0.06 ± 0.03 |
| BASO (K/μl) | 0.00 ± 0.00 | 0.00 ± 0.00 | 0.00 ± 0.00 | 0.00 ± 0.00 | 0.00 ± 0.00 | 0.00 ± 0.00 | 0.00 ± 0.00 | 0.00 ± 0.00 |

WBC = White blood cells; RBC = Red blood cells; Hb = Hemoglobin; HCT = Hematocrits; MCV = Mean corpuscular volume; MCH = Mean corpuscular hemoglobin; MCHC = Mean corpuscular hemoglobin concentration; RDW = Red cell distribution width; PLT = Platelets; MPV = Mean platelet volume; NEU = Neutrophils; LYO = Lymphocytes; MONO = Monocytes; EOS = Eosinophils; BASO = Basophils.

*: Significant difference from the control group of female rats ($P < 0.05$). Results are given as mean S.D., n = 10.

**Table 2.** Effects of ammonium persulfate on parameters of blood chemistry in rats

| Blood chemistry parameters | Ammonium persulfate (mg/kg) Female (n = 10) | | | | Ammonium persulfate (mg/kg) Male (n = 10) | | | |
|---|---|---|---|---|---|---|---|---|
| | 0 | 5 | 20 | 80 | 0 | 5 | 20 | 80 |
| ALB (g/dl) | 2.97 ± 0.23 | 2.87 ± 0.18 | 2.86 ± 0.20 | 2.94 ± 0.20 | 2.26 ± 0.08 | 2.34 ± 0.14 | 2.36 ± 0.10 | 2.28 ± 0.19 |
| ALP (IU/l) | 149.00 ± 30.22 | 165.50 ± 47.37 | 174.40 ± 73.12 | 148.40 ± 59.77 | 303.40 ± 54.04 | 304.90 ± 57.08 | 266.10 ± 46.52 | 208.30* ± 31.07 |
| CA (mg/dl) | 11.00 ± 0.40 | 10.76 ± 0.46 | 10.90 ± 0.41 | 10.94 ± 0.32 | 10.72 ± 0.41 | 11.03 ± 0.31 | 10.88 ± 0.42 | 10.59 ± 0.61 |
| CHO (mg/dl) | 100.80 ± 29.96 | 103.90 ± 28.52 | 95.50 ± 15.62 | 101.70 ± 18.02 | 86.80 ± 16.54 | 78.40 ± 20.81 | 91.60 ± 21.44 | 77.10 ± 20.93 |
| CRE (mg/dl) | 0.89 ± 0.09 | 0.88 ± 0.12 | 0.91 ± 0.07 | 0.84 ± 0.10 | 0.67 ± 0.09 | 0.70 ± 0.07 | 0.74 ± 0.11 | 0.61 ± 0.23 |
| GGT (IU/l) | 0.00 ± 0.00 | 0.00 ± 0.00 | 0.00 ± 0.00 | 0.00 ± 0.00 | 0.60 ± 0.52 | 0.40 ± 0.52 | 0.50 ± 0.53 | 0.40 ± 0.70 |
| GLU (mg/dl) | 137.60 ± 35.18 | 126.00 ± 35.99 | 133.80 ± 32.26 | 145.00 ± 30.58 | 206.40 ± 40.40 | 202.30 ± 28.34 | 198.70 ± 49.58 | 184.90 ± 48.35 |
| AST (IU/l) | 90.50 ± 28.85 | 98.80 ± 36.58 | 78.70 ± 10.34 | 74.60 ± 9.75 | 115.10 ± 75.88 | 107.90 ± 36.21 | 78.50 ± 11.99 | 83.30 ± 19.24 |
| ALT (IU/l) | 38.80 ± 8.07 | 41.30 ± 17.55 | 34.50 ± 6.47 | 34.00 ± 7.96 | 67.60 ± 59.76 | 53.50 ± 20.87 | 39.20 ± 6.27 | 43.00 ± 10.74 |
| LDH (IU/l) | 147.50 ± 100.35 | 265.30* ± 144.97 | 139.10 ± 103.94 | 96.60 ± 63.97 | 166.30 ± 98.60 | 204.00 ± 170.21 | 177.80 ± 123.62 | 170.60 ± 144.71 |
| MG (mg/dl) | 2.59 ± 0.16 | 2.76 ± 0.29 | 2.67 ± 0.22 | 2.70 ± 0.18 | 2.56 ± 0.35 | 2.74 ± 0.36 | 2.76 ± 0.31 | 2.60 ± 0.36 |
| TP (g/dl) | 6.77 ± 0.36 | 6.69 ± 0.31 | 6.70 ± 0.36 | 6.60 ± 0.37 | 6.02 ± 0.19 | 6.22 ± 0.31 | 6.32* ± 0.25 | 5.96 ± 0.34 |
| UA (mg/dl) | 1.77 ± 0.36 | 2.06 ± 0.46 | 2.58 ± 0.82 | 2.63 ± 1.22 | 2.93 ± 1.23 | 3.48 ± 1.37 | 3.26 ± 1.33 | 2.61 ± 1.28 |
| BUN (mg/dl) | 17.97 ± 3.16 | 17.48 ± 3.29 | 17.93 ± 1.15 | 15.03 ± 3.29 | 15.39 ± 1.59 | 15.33 ± 1.67 | 15.29 ± 2.04 | 13.01* ± 2.03 |
| T-BIL (mg/dl) | 0.08 ± 0.03 | 0.09 ± 0.04 | 0.08 ± 0.02 | 0.08 ± 0.02 | 0.05 ± 0.03 | 0.04 ± 0.02 | 0.04 ± 0.02 | 0.04 ± 0.01 |
| IP (mg/dl) | 8.48 ± 1.00 | 9.46 ± 1.47 | 9.24 ± 1.55 | 9.03 ± 1.38 | 9.04 ± 0.76 | 10.29 ± 2.05 | 16.83 ± 23.63 | 8.82 ± 1.13 |
| TG (mg/dl) | 42.10 ± 22.37 | 32.70 ± 17.96 | 37.60 ± 27.09 | 35.10 ± 8.88 | 41.40 ± 14.37 | 42.30 ± 22.92 | 51.60 ± 30.78 | 44.40 ± 16.31 |
| CPK (U/l) | 121.20 ± 48.62 | 168.00 ± 64.33 | 126.40 ± 48.26 | 103.3 ± 30.67 | 158.40 ± 38.02 | 187.60 ± 111.21 | 151.80 ± 55.19 | 150.90 ± 66.74 |
| Na (mmol/l) | 143.00 ± 1.41 | 143.20 ± 1.99 | 144.40 ± 2.27 | 143.40 ± 2.41 | 145.40 ± 1.07 | 146.20 ± 1.32 | 145.70 ± 1.16 | 145.80 ± 1.87 |
| K (mmol/l) | 5.07 ± 1.0 0 | 4.67 ± 1.25 | 4.86 ± 0.84 | 5.46 ± 0.90 | 4.87 ± 0.55 | 5.39 ± 0.69 | 5.00 ± 0.86 | 5.13 ± 0.77 |
| Cl (mmol/l) | 104.60 ± 1.43 | 104.70 ± 1.16 | 104.90 ± 2.56 | 105.00 ± 1.83 | 104.30 ± 1.25 | 104.20 ± 1.55 | 103.70 ± 1.34 | 105.70* ± 1.49 |

ALB = Albumin; ALP = Alkaline phosphatase; CA = Calcium; CHO = Total cholesterol; CRE = Creatinine; GGT = Gamma glutamyl transpeptidase; GLU = Glucose; AST = Aspartate aminotransferase; ALT = Alanine aminotransferase; LDH = Lactate dehydrogenase; MG = Magnesium; TP = Total protein; UA = Uric acid; BUN = Blood urea nitrogen; T-BIL = Total bilirubin; IP = Inorganic phosphorus; TG = Triglyceride; CPK = Creatine phosphokinase; Na = Sodium; K = Potassium; Cl = Chloride.
*: Significant difference from the control group of female rats ($P < 0.05$). Results are given as mean S.D., n = 10.

**Table 3.** Absolute and mean relative organ weights in rats administered ammonium persulfate for 90 days

| Ammonium persulfate (mg/kg) | Brain (g) | Liver (g) | Kidney left (g) | Adrenals left (g) | Adrenals right (g) | Testis/ovary left (g) | Testis/ovary right (g) |
|---|---|---|---|---|---|---|---|
| Female | | | | | | | |
| 0 | 1.92 ± 0.06 | 7.34 ± 0.63 | 0.88 ± 0.09 | 0.031 ± 0.005 | 0.031 ± 0.006 | 0.06 ± 0.01 | 0.06 ± 0.01 |
| | (0.67 ± 0.04) | (2.56 ± 0.15) | (0.31 ± 0.03) | (0.011 ± 0.002) | (0.011 ± 0.002) | (0.02 ± 0.00) | (0.02 ± 0.00) |
| 5 | 2.01 ± 0.06 | 7.80 ± 0.89 | 0.91 ± 0.10 | 0.039 ± 0.009 | 0.041 ± 0.005* | 0.06 ± 0.01 | 0.06 ± 0.01 |
| | (0.67 ± 0.06) | (2.59 ± 0.12) | (0.30 ± 0.03) | (0.014 ± 0.002) | (0.014 ± 0.002) | (0.02 ± 0.00) | (0.02 ± 0.00) |
| 20 | 1.95 ± 0.10 | 7.65 ± 1.08 | 0.87 ± 0.07 | 0.038 ± 0.008 | 0.036 ± 0.006 | 0.07 ± 0.01* | 0.06 ± 0.01 |
| | (0.69 ± 0.08) | (2.67 ± 0.18) | (0.31 ± 0.03) | (0.013 ± 0.003) | (0.013 ± 0.002) | (0.02 ± 0.00) | (0.02 ± 0.00) |
| 80 | 1.99 ± 0.08 | 7.51 ± 0.73 | 0.86 ± 0.11 | 0.035 ± 0.007 | 0.036 ± 0.007 | 0.06 ± 0.01 | 0.06 ± 0.01 |
| | (0.71 ± 0.07) | (2.67 ± 0.13) | (0.31 ± 0.03) | (0.012 ± 0.003) | (0.013 ± 0.003) | (0.02 ± 0.00) | (0.02 ± 0.00) |
| Male | | | | | | | |
| 0 | 2.10 ± 0.12 | 14.42 ± 2.23 | 1.63 ± 0.22 | 0.036 ± 0.018 | 0.036 ± 0.018 | 1.67 ± 0.23 | 1.67 ± 0.22 |
| | (0.37 ± 0.02) | (2.53 ± 0.21) | (0.29 ± 0.03) | (0.006 ± 0.003) | (0.006 ± 0.003) | (0.29 ± 0.04) | (0.29 ± 0.04) |
| 5 | 2.17 ± 0.11 | 13.80 ± 1.36 | 1.60 ± 0.11 | 0.045 ± 0.032 | 0.045 ± 0.033 | 1.74 ± 0.17 | 1.73 ± 0.19 |
| | (0.41 ± 0.04) | (2.60 ± 0.19) | (0.31 ± 0.03) | (0.009 ± 0.006) | (0.009 ± 0.006) | (0.33 ± 0.05) | (0.33 ± 0.05) |
| 20 | 2.15 ± 0.10 | 15.62 ± 3.20 | 1.68 ± 0.18 | 0.029 ± 0.006 | 0.029 ± 0.005 | 1.78 ± 0.14 | 1.78 ± 0.15 |
| | (0.39 ± 0.05) | (2.80 ± 0.34) | (0.30 ± 0.03) | (0.005 ± 0.001) | (0.005 ± 0.001) | (0.32 ± 0.03) | (0.33 ± 0.03) |
| 80 | 2.15 ± 0.07 | 14.65 ± 2.49 | 1.71 ± 0.26 | 0.051 ± 0.036 | 0.047 ± 0.036 | 1.75 ± 0.14 | 1.77 ± 0.55 |
| | (0.39 ± 0.04) | (2.63 ± 0.29) | (0.31 ± 0.04) | (0.009 ± 0.007) | (0.009 ± 0.007) | (0.32 ± 0.03) | (0.32 ± 0.04) |

[a]Results are given as a mean ± S.D., n = 10.
[b]Absolute organ weight (g).
[c]Mean relative organ weight (g/100 g body weight).
[d]Significant difference from the control group of female rats ($P < 0.05$).

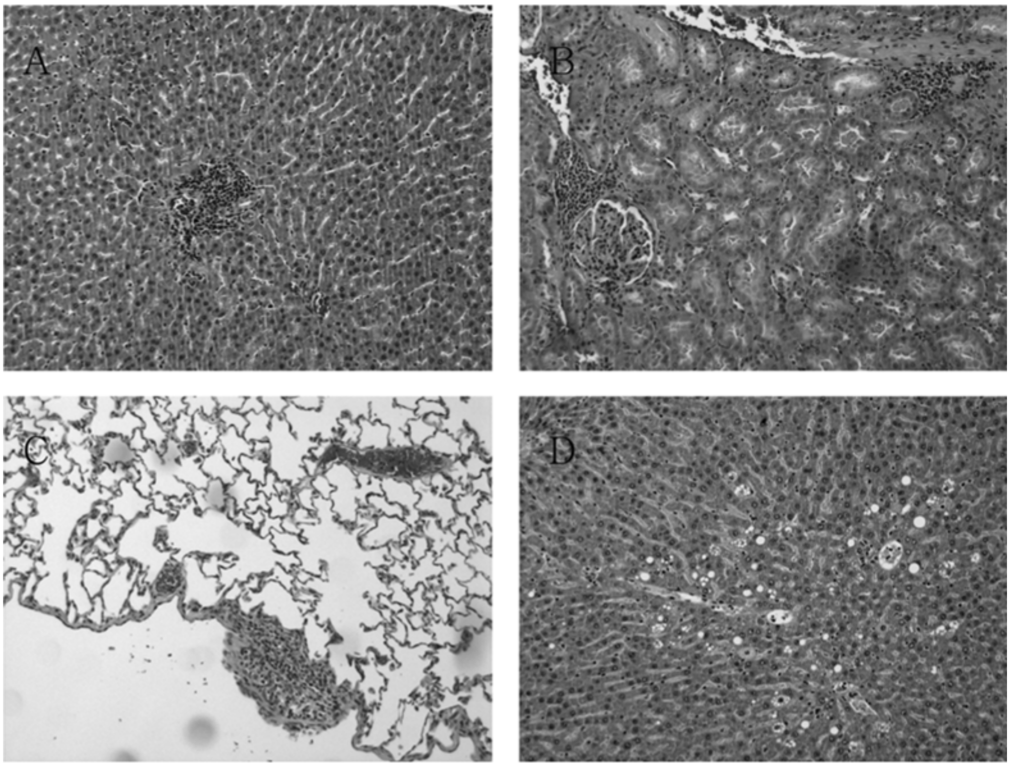

**Fig. 3.** Histopathological changes of rats with administered with ammonium persulfate. 80 mg/kg group the male rats appear focal inflammatory cell infiltration (A) lung, (B) liver and (C) kidney. 20 mg/kg group, the female rats appear diffuse vaoculation in the liver (D). (H-E, × 200).

blood analysis and histopathological examination. There were significant changes about food consumption, organ weight, some parameters (monocyte, total protein, LDH, *etc.*) on blood chemistry and inflammation, however, considered as normal reference limit range (Matsuzawa *et al.*, 2000; Smyth, 1969) and non-administration-related phenomenon. Therefore, the no observed adverse effect level (NOAEL) values for sub-chronic test was determined 80 mg/kg b.w. or more than that.

Previous reported had shown the acute and sub-chronic inhalation toxicity tests of ammonium persulfate, $LD_{50}$ value for acute was 2.95 mg/l or more, or 520 mg/l and the NOAEL value was 1.3 mg/m$^3$ (Budavari *et al.*, 1989; Last *et al.*, 1982). Similarly test for 28-day oral toxicity test had shown the NOAEL value was 41.4 mg/kg (Pang, 2001). Skin and eye irritation, carcinogenic, reproductive and genetic toxicity test had also studied by other groups, but those revealed that no toxicity of test substance (CTFA, 1994; Huntington Research Center, 1997).

An interesting observation in this study was that clinical signs of digestive system including diarrhea, discoloration of alimentary tracts. And decreased level of BUN considered as one of renal toxicity indicating parameter in high dose male group. Further study for these two matters should be needed.

In conclusion, the present study showed that, acute and sub-chronic oral administration with ammonium persulfate, rats exhibited no administration-related toxicological and histopathological abnormalities. This provides confidence that any potential accumulation of active constituents resulting from ammonium persulfate in public and industrial fields environmental disclosure does not lead to toxicity. The doses of ammonium persulfate tested in rats were far greater than any doses anticipated for human exposure. It is likely that no acute or sub-chronic toxicological risk would occur with low doses of ammonium persulfate commonly exposed by humans. However, this extrapolation should be drawn with caution since the human risk is not possible to assess from the present study.

## ACKNOWLEDGMENTS

Thus study was supported by KOSHA (Korea Occupational Safety and Health Agency, Grant No. 2008-94-1162).
